# Comparison of multi-class and fusion of multiple single-class SegNet model for mapping karst wetland vegetation using UAV images

**DOI:** 10.1038/s41598-022-17620-2

**Published:** 2022-08-02

**Authors:** Tengfang Deng, Bolin Fu, Man Liu, Hongchang He, Donglin Fan, Lilong Li, Liangke Huang, Ertao Gao

**Affiliations:** College of Geomatics and Geoinformation, Guilin University of Technology, No.319 Yanshan Street, Guilin, 541006 China

**Keywords:** Limnology, Wetlands ecology

## Abstract

Wetland vegetation classification using deep learning algorithm and unmanned aerial vehicle (UAV) images have attracted increased attentions. However, there exist several challenges in mapping karst wetland vegetation due to its fragmentation, intersection, and high heterogeneity of vegetation patches. This study proposed a novel approach to classify karst vegetation in Huixian National Wetland Park, the largest karst wetland in China by fusing single-class SegNet classification using the maximum probability algorithm. A new optimized post-classification algorithm was developed to eliminate the stitching traces caused by SegNet model prediction. This paper evaluated the effect of multi-class and fusion of multiple single-class SegNet models with different EPOCH values on mapping karst vegetation using UAV images. Finally, this paper carried out a comparison of classification accuracies between object-based Random Forest (RF) and fusion of single-class SegNet models. The specific conclusions of this paper include the followings: (1) fusion of four single-class SegNet models produced better classification for karst wetland vegetation than multi-class SegNet model, and achieved the highest overall accuracy of 87.34%; (2) the optimized post-classification algorithm improved classification accuracy of SegNet model by eliminating splicing traces; (3) classification performance of single-class SegNet model outperformed multi-class SegNet model, and improved classification accuracy (F1-Score) ranging from 10 to 25%; (4) Fusion of single-class SegNet models and object-based RF classifier both produced good classifications for karst wetland vegetation, and achieved over 87% overall accuracy.

## Introduction

Wetlands are some of the most productive habitat and ecosystem types on the planet^1,2^. Karst wetland has been as a unique type of underground wetland system in the Ramsar classification, with or without a surface water component, commonly associated with caves or other underground cavities. Karst wetlands have played important environmental adjustment roles, such as maintain water sources, purify water quality etc., and possesses unique ecological benefits and research values^3–5^. The Huixian Karst National Wetland Park (HKWP) of the study area is in the core of the East Asia karst area, the third-largest karst area in the world and the largest karst wetland in China, and possesses unique ecological and landscape values that help promote Guilin city as a world tourist attraction. However, karst wetlands in the HKWP have been seriously damaged under the influence of human activities, which have already appeared area reduction, functional degradation and biodiversity loss. Vegetation is an important indicator of wetland environmental change^6^. Therefore, mapping spatial distribution of vegetation is essential for protecting, managing and restoring karst wetlands of HKWP.

Current researches for karst wetlands mainly focus on monitoring the organic matter or microorganism content in the soil to evaluate the pollution status of karst wetland^7,8^,including monitoring chemical and isotopic evolution of groundwater in an evaporite karst plateau (including wetland areas and saline to hyper-saline springs) located at southern Spain^9^. Some scholars constructed mass balances of both calcium and phosphorus for two watersheds in Big Cypress National Preserve in southwest Florida (USA) to evaluate the time scales over which its striking landscape pattern developed^10^. Several studies have analyzed the distribution and change of karst vegetation using field survey and field sampling^11,12^. However, in situ floristic mapping and data collection usually are expensive, labor intensive and inaccessible or even dangerous to human beings in the karst wetlands.

Remote sensing techniques have been used to classify vegetation in marsh, estuary and coastal wetlands, and other wetland types^13–16^. Lane et al. explored and evaluated the utility of a newly launched high-resolution, eight-band Worldview-2 images for classifying freshwater deltaic wetland vegetation and aquatic habitats in the Selenga River Delta of Lake Baikal^17^. Betbeder et al. classified wetland vegetation formations at a 1:10,000 scale using eight dual-polarization TerraSAR-X images acquired in 2013 during dry and wet seasons^18^. Franklin et al. distinguished circumboreal Canadian bogs and fens by differences in soil, hydrology, vegetation, and morphological features by combining Radarsat-2 full-polarimetric synthetic aperture radar (SAR) data and Landsat-8 Operational Land Imager (OLI) multi-spectral images^19^. The aforementioned studies confirmed that remote sensing provides a practical means for data collection and vegetation mapping in the wetland. Unmanned aerial vehicles (UAVs) offer a cost-effective, flexible approach to acquire the ultra-high spatial and temporal resolution images for discriminating and mapping salt marsh species (2020) and mangrove species^20,21^. However, the composition and spatial distribution patterns of karst wetland vegetation have great different from other wetland types, due to its unique hydrogeological condition. In addition, the vegetation patches are usually very small, and located around karst rivers and lakes. Classification of karst wetland vegetation still faces great challenges with a large number of fragmentations, intersection, and high heterogeneity of vegetation patches.

Shallow machine learning algorithms, such as maximum likelihood, k-nearest neighbor (KNN), support vector machine (SVM), decision tree and random forest (RF) classifiers have long been the main methods for discriminating wetland vegetation^22–26^. However, these algorithms usually spend a lot of time and effort to select optimal input features from raw data. Transfer learning produced by shallow machine learning algorithms also has a large learning cost. However, deep learning algorithms have provided an effective method to automatically learn and identify features from large amounts of raw data with little or no preprocessing^27^. Several deep learning architectures, such as convolutional neural network (CNN), fully convolutional network (FCN), SegNet, PSPNet and DeepLabV3 plus networks have achieved state-of-the-art performance in land cover classification^28^ and water body extraction^29^. These studies confirmed that a powerful CNN requires a large amount of training data^30,31^. However, the important semantic labeling data of training dataset for deep-learning model has been currently produced by visual interpretation, which usually consumes a lot of time and labor intensive. This paper presents an approach to create semantic labeling data by combing object-based RF classification with visual interpretation of UAV images. In addition, there exist many challenges in identifying multi-class land cover types using deep learning algorithms, such as a large dataset and for a lot of time training, class imbalance, etc. In order to solve some of these problems, we propose a novel single class deep learning approach for classifying karst wetland vegetation, and demonstrated that the feasibility of fusing single-class SegNet classification for karst wetland vegetation mapping using the maximum probability approach.

The main original contributions of this study are that: (1) we propose a novel classification technique for mapping karst wetland vegetation by fusing multiple single-class SegNet models using the maximum probability(MP), and evaluated the performance of multi-class SegNet model and fusion of single-class SegNet models on karst wetland vegetation mapping in Huixian National Wetland Park;(2) this paper present a new approach to create semantic labeling dataset by combining object-based RF classification model with visual interpretation; (3) we developed an optimized post-classification algorithm to eliminate the splicing traces caused by model prediction of SegNet network; (4)we explored the effects of EPOCH values and texture feature on karst wetland vegetation classification.

## Study area and data source

### Study area

Huixian National Wetland Park (25°01′30″N ~ 25°11′15″N, 110°08′15″E ~ 110°18′00″E) is located in Guilin city, Guangxi province, southeast China. The total length of wetland park is about six kilometers from east to west. The total width is about 2.8 km from north to south. The Huixian wetland is the most evocative and largest karst wetland in China and even the world's low-lying and low-altitude karst areas. This wetland feature and its surroundings are extremely rare in the global peak forest karst plain features and are typically wetlands of significant research values^32,33^. Since the 1950s, the original karst wetlands have been continuously undermined, due to human activities and the lack of effective management and protection. The water body of wetland has gradually been shrinking, and extensive karst wetlands were reclaimed as paddy field and fish ponds, which have resulted in the decrease of biodiversity. In addition, invasive species, such as *water hyacinth*, have increasingly threatened wild plant species in the karst wetlands.

**Figure 1:**
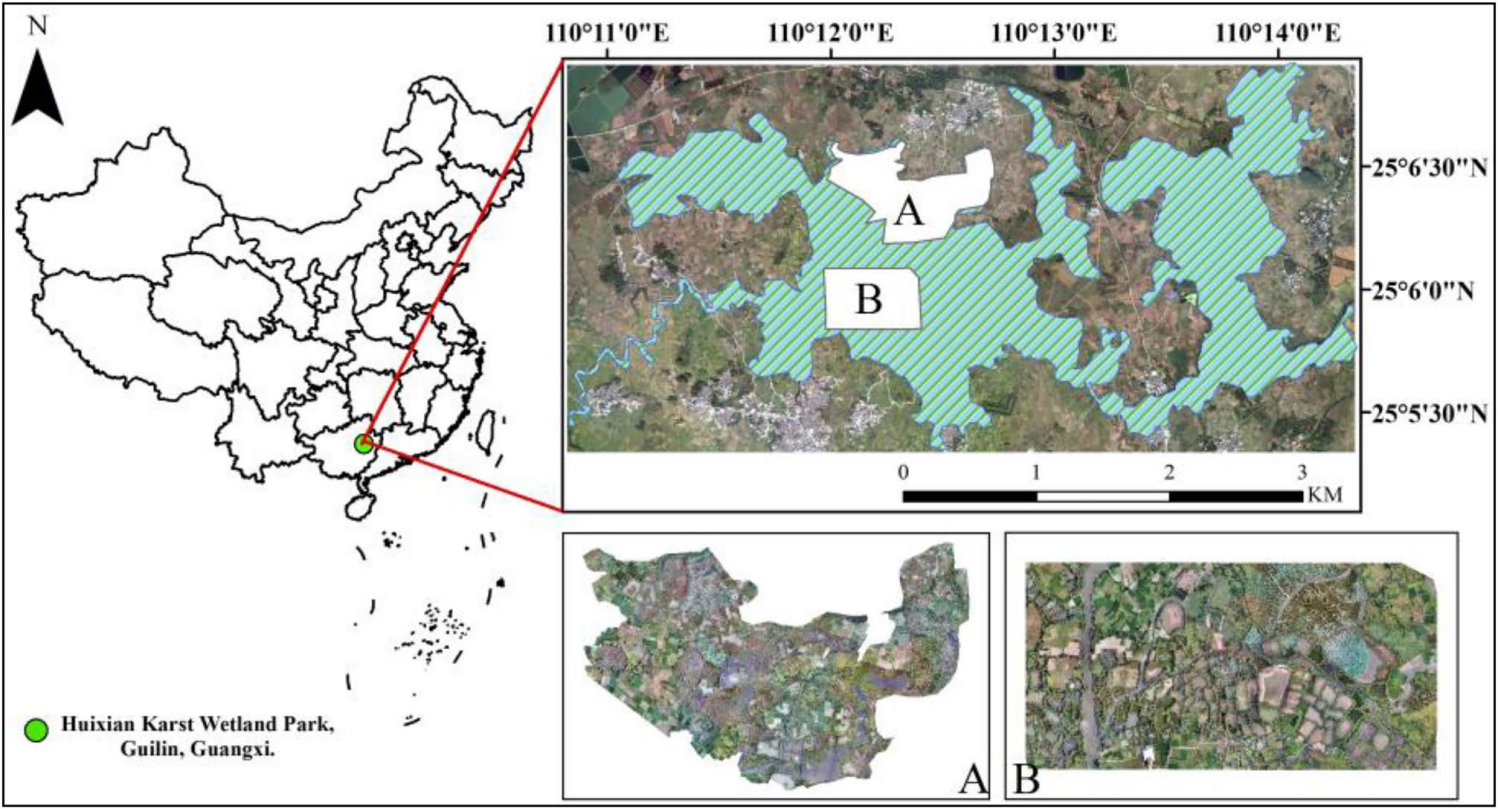
The study area and the location (Area A and B are UAV ortho-images collected by B. F. using the DJI Phantom 4 Pro professional V2.0 drone). ArcGIS 10.6 software (http://www.esri.com/software/arcgis) was used to process and map the data by first author (T. D.) and corresponding author (B. F.). The Map of China (left) is credited to the SinoMaps Press, previously known as China Cartographic Publishing House.

Through field investigation and analysis of the basic characteristics of Huixian National Wetland Park, the core areas A and B of Huixian National Wetland Park were selected as the test area (Fig. 1), which covered the original and complete karst wetland vegetation types, such as *lotus*, *water hyacinth*, *bamboo* and *linden*. The ecological appearance is less damaged by human activities. Wetland vegetation usually surrounded by karst rivers and lakes.

### UAV image collections and preprocessing

The original UAV images were collected using a DJI Phantom 4 Pro professional V2.0 drone with a 1-inch 20MP CMOS camera from 10:00 am to 3:00 pm (UTC/GMT + 8.00) during July 10 ~ 20, 2018. During the acquisition process, the weather was sunny, windless, and the field of vision was good. The flight path was automatically generated using the ground station software (DJI GS PRO), where the heading overlap rate was 80%, the side overlap rate was 75%. The flight height and field of view (FOV) was 90 m and default value 84°, respectively. Ground Control Points (GCPs) were obtained using a Trimble R6 GPS-RTK with positioning accuracy ± 2 cm (Trimble, CA, USA) for georeferencing and image stitching during UAV images processing.

This paper used the Pix4D Mapper software to process the UAV original images for obtaining the Digital Orthophoto images (DOM) of the study area. The main pre-processing steps included that: (1) importing of original aerial image and POS data, including latitude and longitude coordinates, flight height, and GCPs; (2) checking images quality, and removing the images with the end overlap of less than 80% and side overlap of less than 70%; (3) automatic image matching from multiple overlapping UAV imagery, aerial triangulation, and block adjustment to generate dense point cloud data; (4) building a TIN triangle network to obtain three-dimensional surface model of the study area; (5) the DOM images of study area produced by the three-dimensional surface model and aerial triangulation parameters. Moreover, this paper used a Seamless Mosaic tool in ENVI 5.4 software for image mosaic and georeferencing.

Plot-based filed measurements were carried out from July 10–25, 2018 in the Huixian National Wetland Park. Vegetation type and geographical location of each 1 × 1 m sample plot were identified, and recorded by the Trimble R6 GPS-RTK. Finally, a total of 612 sample data were obtained, which were divided into 8 categories, including *karst water*, *karst grass*, *karst *broad*-leaved forest*, and *karst aquatic flora*. The sample data was randomly divided into two parts: 2/3 for training data and 1/3 for test data using the RStudio software (Table 1). The training data was utilized to produce object-based RF classification for karst wetland vegetation. The testing data were used to verify the classification accuracy of object-based RF and SegNet models.

**Table 1:** Summary of the training and testing samples derived from vegetation plots.

Regions	Types	*Karst water*	*Karst grass*	*Karst broad-leaved forest*	*Karst aquatic flora*	*Other types*	Total
Area A	Training	34	58	32	52	22	198
	Testing	20	20	20	20	20	100
Area B	Training	42	48	36	60	28	214
	Testing	20	20	20	20	20	100

## Methods

**Figure 2:**
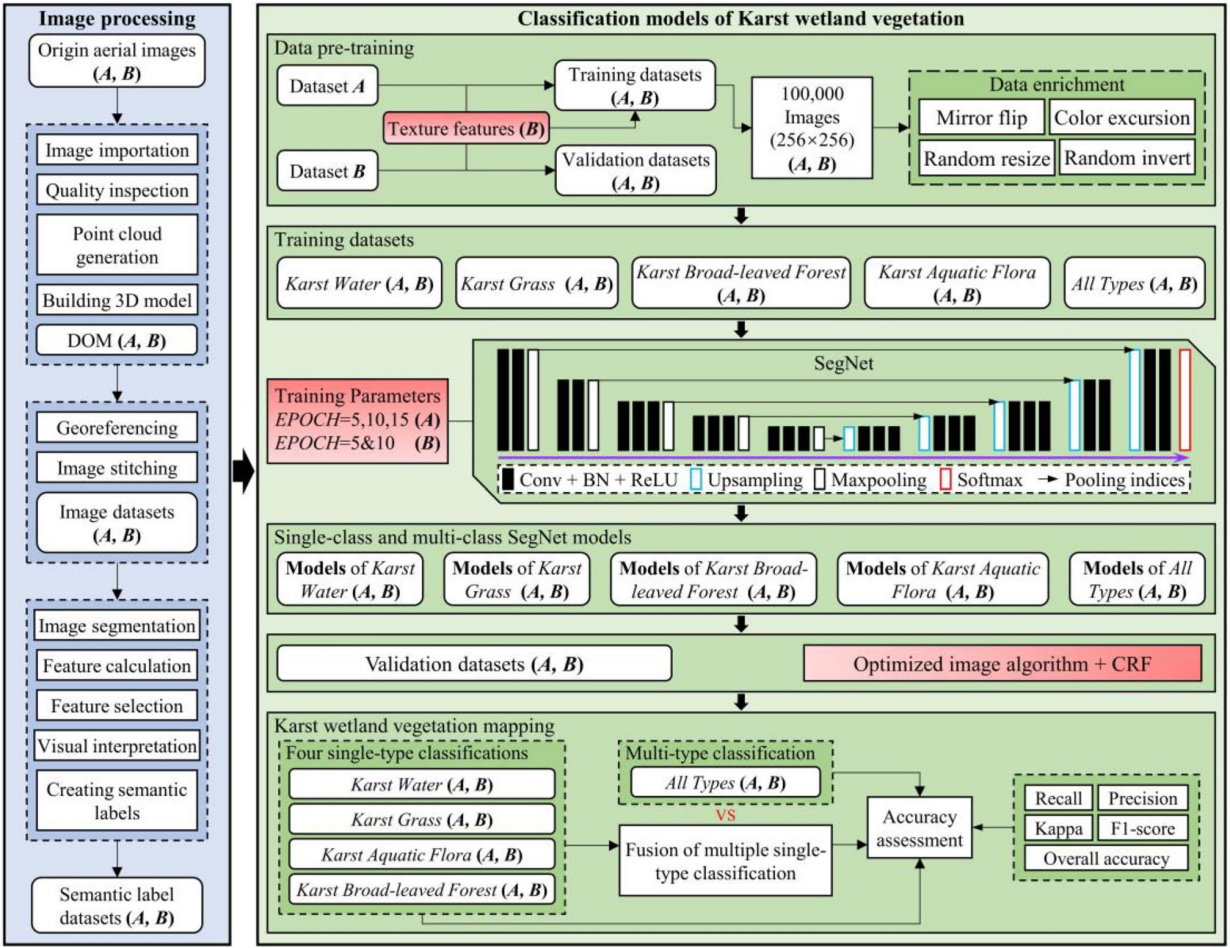
Flowchart of classifying karst wetland vegetation using SegNet model.

To achieve high-precision classifications of karst wetland vegetation, this study built multi-class SegNet model and fusion of multiple single-class SegNet model with different EPOCH values and UAV images. The flowchart of this study mainly included three parts (Fig. 2): (1) UAV images collection and preprocessing. (2) combination of object-based RF classifications and visual interpretation was used to produce semantic labeling dataset. (3) construction of multi-class SegNet model and fusion of four single-class SegNet models with different EPOCH values (5, 10 and 15) and texture features. (4) development of post-classification algorithm and conditional random field (CRF) for optimizing SegNet-based classification results. (5) evaluating the performance of multi-class SegNet model and fusion of multiple single-class SegNet models on mapping karst wetland vegetation.

### Creating semantic labeling dataset using object-based RF classifications and visual interpretation

**Figure 3:**
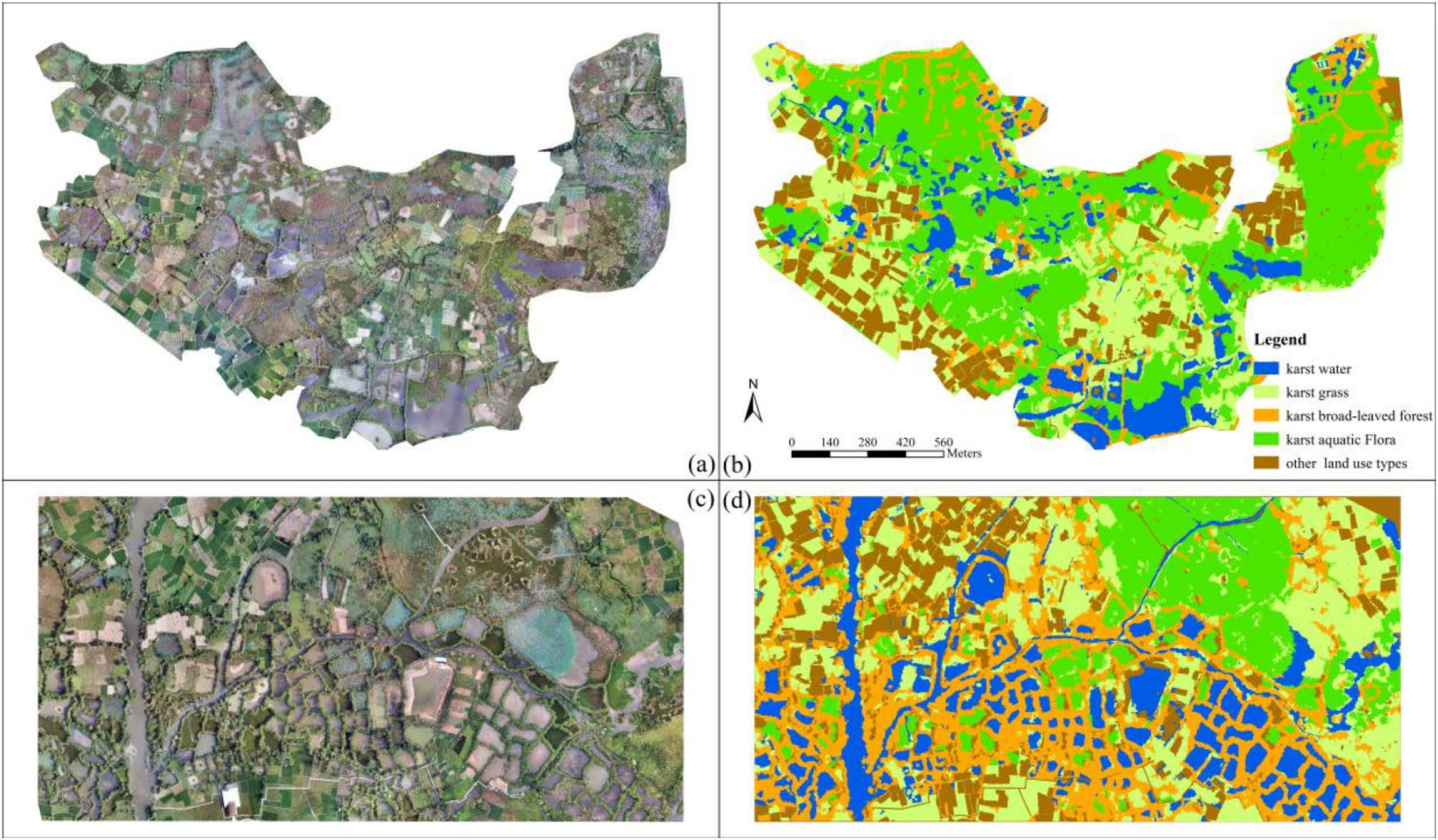
The semantic labeling dataset for SegNet classification (**a**) DOM image of area A. (**b**) semantic labeling dataset of Area A, 10,550*5320 pixels. (**c**) DOM image of area B. (**d**) semantic labeling dataset of area B, 11,290*7176 pixels.). ArcGIS 10.6 software (http://www.esri.com/software/arcgis) was used to process and map the data by first author (T. D.) and corresponding author (B. F.). The DOM images and semantic labeling dataset were collected and created by T. D. and B. F.

RF is a machine learning algorithm^34^ with the advantages of high classification accuracy, strong generalization ability, and providing variable importance estimation. It has been used for wetland vegetation classification^35,36^. In this study, semantic labeling dataset was created by combining object-based RF classifications and visual interpretation. We classified karst wetland vegetation using the object-based RF algorithm, then the vegetation patches misclassified by RF model were corrected by visual interpretation with the field measurements. The semantic labeling dataset for SegNet-based classifications and original UAV DOM images of area A and B were displayed in Fig. 3.

There are three main steps for object-based RF classification for karst wetland vegetation: image segmentation, feature selection, model training and prediction. The specific process was as follows:

(1) Image segmentation. This paper used multi-scale segmentation algorithm to segment the UAV images. Three segmentation parameters, i.e., color/shape weights, smoothness/compactness weights and a scale parameter were considered. An automated segmentation scale parameter selection tool (ESP2) based on secondary development of eCognition Developer software was used to determine the optimal scale parameters of Area A (120) and Area B (127) by 500 iterative segmentations. We tried different combinations of color/shape weight and smoothness/compactness weight for segmenting UAV image, such as 0.1/0.9, 0.3/0.7, 0.5/0.5, and 0.7/0.3. The optimal combination was 0.1/0.9 and 0.5/0.5, respectively.

(2) Feature selection. This paper comprehensively considers five types of image features, including spectral feature, texture feature, shape features, and context variables. All image features were calculated using eCognition 9.4 software. The grey-level co-occurrence matrix (GLCM) was used to calculated the textural features, including *mean, variance, homogeneity, contrast, dissimilarity, entropy, *etc*.* The Recursive feature elimination (RFE) algorithm was used to select optimal input features of Area A (49) and B (56) for RF classification.

(3) Classification model training and prediction. The tuning parameters of object-based RF classification included the max number of trees (*ntree*), the number of split variables (*mtry*). In this study, the optimal parameters of ntree and mtry were set 1200 and 8, respectively. The RF-based model training and classification for karst wetland vegetation were carried out using the randomForest package.

### SegNet-based classification of Karst wetland

SegNet architecture is designed to be an efficient architecture for pixel-wise semantic segmentation, which mainly composed of encoder network, decoder network, and pixel-wise classification layer^37^. The layers are followed by batch normalization^38^ and the rectified linear unit (ReLU)activation function. It provides a good balance between accuracy and computational cost. In addition, symmetrical architecture and combination of the pooling/upsampling of SegNet architecture are very effective for precise re-localization of features^39–42^, which is intuitively crucial for remote sensing image classification.

Previous studies reported that as the number of iterations increases, the loss function of DeeplabV3 plus decreased rapidly and gradually stabilized, and the classification accuracy of model is rapidly improved and stabilized^43^. Textural features can improve the spectral separability of wetland vegetation^44^. In order to construct a high-precision SegNet model for mapping karst wetland vegetation, this paper added the textural features into the input image features, and developed four single-class and two multi-class SegNet models with different EPOCH values, which were described as shown in Table 2. An optimized image algorithm and conditional random field (CRF) algorithm were used to conduct post-processing of SegNet-based classifications in this study.

**Table 2:** Description of the SegNet models with different EPOCH values.

Models	Descriptions
*Model-a*	Single-class SegNet model of *karst aquatic flora* with EPOCH 5,10 or 15
*Model-b*	Single-class SegNet model of *broad-leaved forest* with EPOCH 5,10 or 15
*Model-g*	Single-class SegNet model of *karst grass* with EPOCH 5,10 or 15
*Model-w*	Single-class SegNet model of *karst water* with EPOCH 5,10 or 15
Multi-class SegNet model	The multi-classification models with EPOCH 5,10 or 15
Multi-class SegNet model _texture feature_	The multi-class SegNet model with texture feature and EPOCH 10 or 15
Fusion of single-class SegNet model _texture feature_	The fusion of four single-class SegNet models with texture feature and EPOCH 10 or 15

#### Creating training dataset for SegNet model

This study attempted to explore the effect of texture features on the SegNet model for classifying karst wetland vegetation. The deep-learning image dataset of karst wetland (Area B) was added texture features from UAV DOM image. The textural feature was calculated from the grey-level co-occurrence matrix (GLCM) with window size 3 × 3, 5 × 5, and 7 × 7 and 64 greyscale quantization levels to obtain the *mean, variance, homogeneity, contrast, dissimilarity, entropy, second moment* and correlation features in ENVI 5.4 software, respectively. The deep-learning image dataset of karst wetland (Areas A and B) is randomly divided into 100,000 image datasets with the size of 256 × 256 pixels, of which 75% was as training datasets and 25% was as testing datasets. The data enhancement of training dataset was executed including randomly flipped along the x-axis or y-axis mirrored or rotated 90º, 180º, 270º, color shifted, randomly scaled and invert operation.

To evaluate the classification performance of SegNet model for mapping karst wetland vegetation, this study constructed four single-class SegNet model for identifying *karst water*, *karst grass*, *karst broad-leaved forest*, and *karst aquatic flora*, respectively. In addition, the paper also established two multi-class SegNet model for mapping all karst wetland vegetation types.

#### SegNet model training and parameter optimization

Parameter optimization is crucial to deep learning algorithm for classification. Model training in this study was performed on a desktop terminal configured with an NVIDIA GeForce GTX 1080 8G and an Intel Core i7 8700 K. The learning rate can control the learning progress of the model. If it is too small that it will cause the model to converge slowly, and if it is too large that it would cause divergence. The initial value of learning rate in this study was set to 0.001. Gamma can control the rate of change of the learning rate, which was set to 0.1 in this study. The momentum plays a role in accelerating convergence, which was set to 0.8 in this paper. Weight decay can adjust the impact of model complexity on the loss function. The weight decay was set to 0.0001. The step size value was set to 1000 times. Due to the limited performance of desktop terminals with the 100,000 training image datasets, the batch-size was set to 8. To achieve better results for SegNet-based classification, according to the previous research^45,46^, this paper sets Optimizer to SGD and Loss Function to category-cross-entropy loss, respectively. This study attempts to explore the influence of EPOCH value on the SegNet model for discriminating karst wetland vegetation. This paper conducted SegNet models training of Area A using the different EPOCH values ranging from 5 to 15. Meanwhile, the SegNet models training of Area B used the EPOCH values from 5 to 10.

#### Optimized image algorithm for SegNet classification

This study classified karst wetland vegetation using SegNet model and segmented sub-image with the size of 256 × 256 pixels. The classifications of each sub-image were spliced into the final result of study area. The trimming image predicts that re-stitching resulted in relatively obvious stitching traces, which significantly decreased the model classification accuracy.

**Figure 4:**
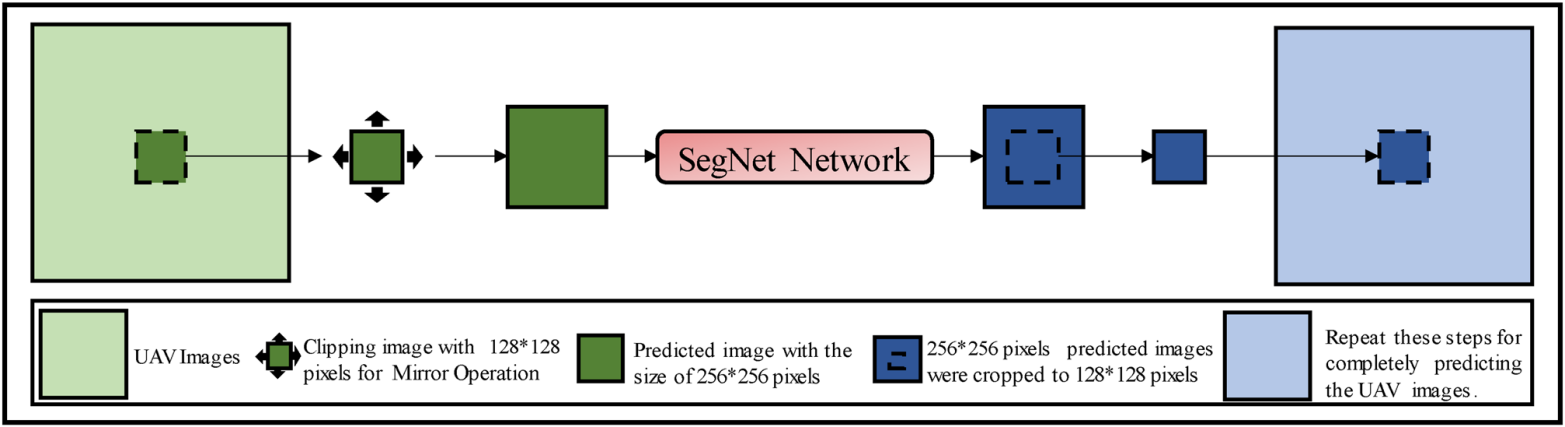
Processing of optimizing image algorithm for post-processing of SegNet model prediction.

To solve this problem, this study designed an optimized image algorithm for post-processing SegNet-based classifications to eliminate the stitching traces. This algorithm integrates edge detection method with filtering algorithm. The core idea of optimizing image algorithms is to expand the region of image datasets, then crop the images after classification, and finally splice them into the final classification results. The specific steps were as follows: (I) cropping UAV image into sub-images with a size of 128 × 128 pixels; (II) expanding the size of sub-images from 128 × 128 pixels to 256 × 256 pixels through the mirror operation; (III) classifying sub-images with a size of 256 × 256 pixels; (IV) trimming the classified 256 × 256 sub-image into 128 × 128 images. The corresponding position of (I) was stitched on the classified sub-image; (V) Finally, repeating the step (I)-(IV) until completely classified the UAV images of Area A and B. The processing of optimizing image algorithm was shown in Fig. 4.

#### Fusion of multiple single-class SegNet models

**Figure 5:**
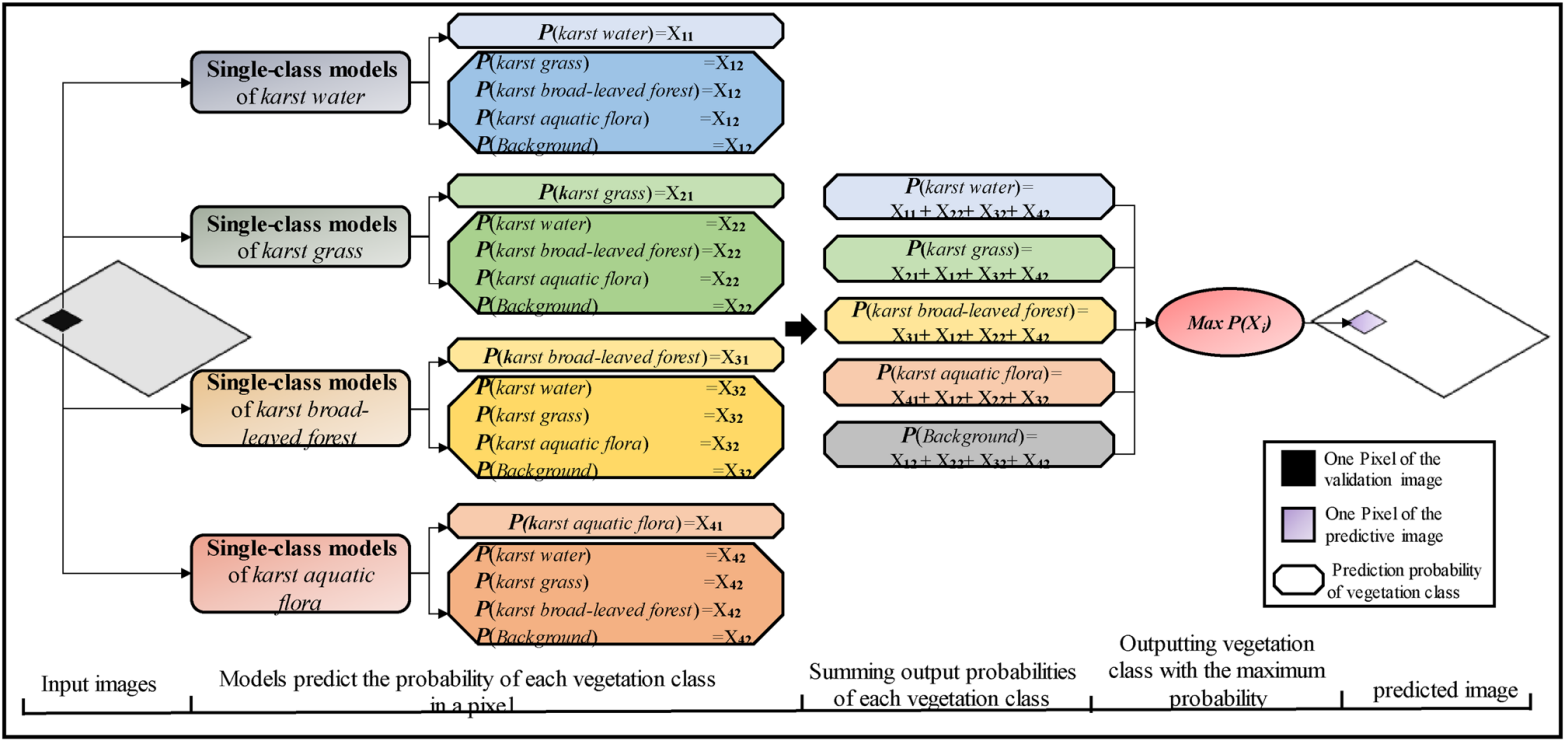
The fusion procedure of multiple single-class SegNet Models.

This study aimed to evaluate the performance of multi-class and fusion of *multiple* single-class SegNet models for classifying karst wetland vegetation. We utilized a method of maximum probability to fuse four single-class SegNet-based classifications into a multi-class classification. The fusion algorithm utilized the Eq. (1) to calculated each single-class SegNet model(*M*_*j*_, *j* = 1,2…4) prediction probability (*P*_*ij*_(*X*_*i*_)) of four vegetation types(*X*_*i*_, *i* = 1,2…4) at each pixel, and summarized of the probability of each vegetation(*P*(*X*_*i*_)) type at a pixel. Finally, the method selected the vegetation type of maximal probability at each pixel as the final multi-class classification results. The specific fusion procedure was shown in Fig. 5.

1 $$\left\{ {\begin{array}{*{20}l} {P(X_{i} ) = \sum\limits_{{j = 1}}^{4} {P_{{ij}} (M_{j} ){\mkern 1mu} i = 1,2, \cdots ,4} } \hfill \\ {P_{{k0}} (X_{i} ) = \mathop {\max }\limits_{{1 \le i \le 4}} P_{i} (X_{i} )} \hfill \\ \end{array} } \right.$$

### Ethics approval and consent to participate

The experimental research on plants (either cultivated or wild), including collection of plant material, complied with institutional, national, or international guidelines.

### Method and ethics approval

The authors declare that they have obtained the permissions from the responsible authority for carrying out the study in the Huixian National Wetland Park. The karst plant samples obtained from national parks.

## Results analysis and accuracy assessment

In this paper, an error matrix, Kappa coefficient, overall accuracy, Recall, F1-score and class-specific producer’s and user’s accuracies were used to evaluate the single-class, multi-class and fusion of multiple single-class SegNet classifications using the testing sample dataset and semantic labeling dataset.

### Single-class SegNet clasifications with different EPOCHs

**Figure 6:**
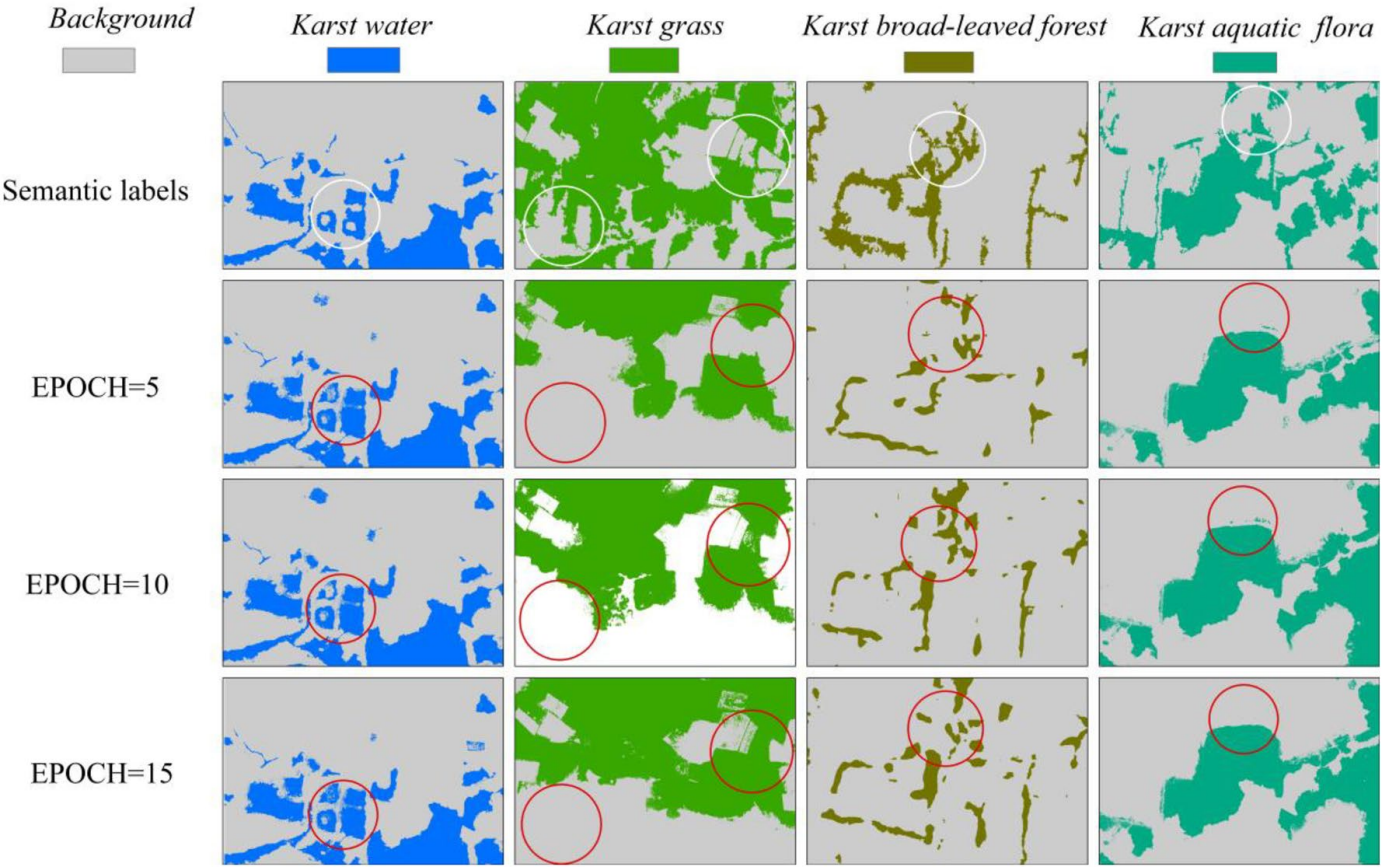
Single-class SegNet-based classifications with different EPOCH values. ArcGIS 10.6 software (http://www.esri.com/software/arcgis) was used to process and map the data by first author (T. D.) and corresponding author (B. F.). The semantic labeling dataset was collected and created by T. D. and B. F.

To evaluate the effect of different EPOCH values on single-class SegNet Models for discriminating karst wetland vegetation, this paper developed four single-class SegNet models with the different EPOCH values (5, 10, 15). The classification results of each model were shown in Fig. 6. The red circle highlighted the mis-classification of karst vegetation type. Comparison of semantic labeling dataset in the white circle, this paper found that (1) the *model-w* with the EPOCH values from 5 to 15 could produce good classification results for *karst water*, and only the isolated trees in the small lakes was falsely identified as *karst water*. In general, with the increase of EPOCH values, the classification of *model-w* for *karst water* was better. (2) Several regions of *karst grass* were not identified using the *model-g*. The classification results of *model-g* with EPCOH 15 were better than that with the other EPCOH values. (3) The *model-b* with three EPCOH values was able to classify *karst broad-leaved forest*, but the whole area of *karst broad-leaved forest* was not completely extracted. (4) The *model-a* with three EPCOH values well depicted *karst* a*quatic flora* vegetation in study area, but the small area of *karst aquatic flora* vegetation was not identified. This study found that the single-class SegNet model with EPCOH 15 produced more visually accurate depictions of karst wetland vegetation.

Table 3 displayed the classification accuracies for four single-class SegNet models, which indicated that the modes with EPOCH 15 was able to achieve higher classification accuracy. The single-class SegNet models achieved over 75% F1-score for all land cover types, but the kappa coefficient of karst grass was below 60%. The *model-a* achieved the highest classification accuracy (F1-score,0.96) when utilizing DOM, followed by *model-w* (F1-score,0.87) and *model-g* (F1-score,0.83). The *karst grass* and *karst broad-leaved forest* were difficult to discern in the single-class SegNet classifications. *Karst broad-leaved forest* was accurately classified in the *model-w* classification achieving 0.91 kappa coefficient. The *model-g* with EPOCH 15 improved F1-score to 0.83 for *karst grass*, an increase of 8% in comparison of using EPOCH 5 or 10. The conclusion in this study was confirmed that the all single-class SegNet models improved above 7% of kappa coefficient when using the EPOCH 15, especially for the *model-g*, which improved 14%. These results demonstrated that the EPOCH values affected the single-class SegNet model for karst vegetation classifications.

**Table 3:** Classification accuracies of single-class SegNet models with different EPOCH values using testing data.

Models	Karst vegetation	EPOCH	Precision	Recall	F1-score	Kappa
*Model-a*	*Karst aquatic flora*	5	0.92	0.93	0.92	0.85
		10	0.92	0.97	0.94	0.88
		15	0.93	0.98	0.96	0.91
*Model-b*	*Karst broad-leaved forest*	5	0.77	0.74	0.75	0.50
		10	0.86	0.74	0.78	0.56
		15	0.84	0.76	0.79	0.58
*Model-g*	*Karst grass*	5	0.93	0.70	0.75	0.52
		10	0.94	0.72	0.78	0.56
		15	0.95	0.78	0.83	0.66
*Model-w*	*Karst water*	5	0.81	0.89	0.84	0.67
		10	0.83	0.89	0.85	0.69
		15	0.85	0.91	0.87	0.74

### Multi-class SegNet classificaitons with different EPOCHs

**Figure 7:**
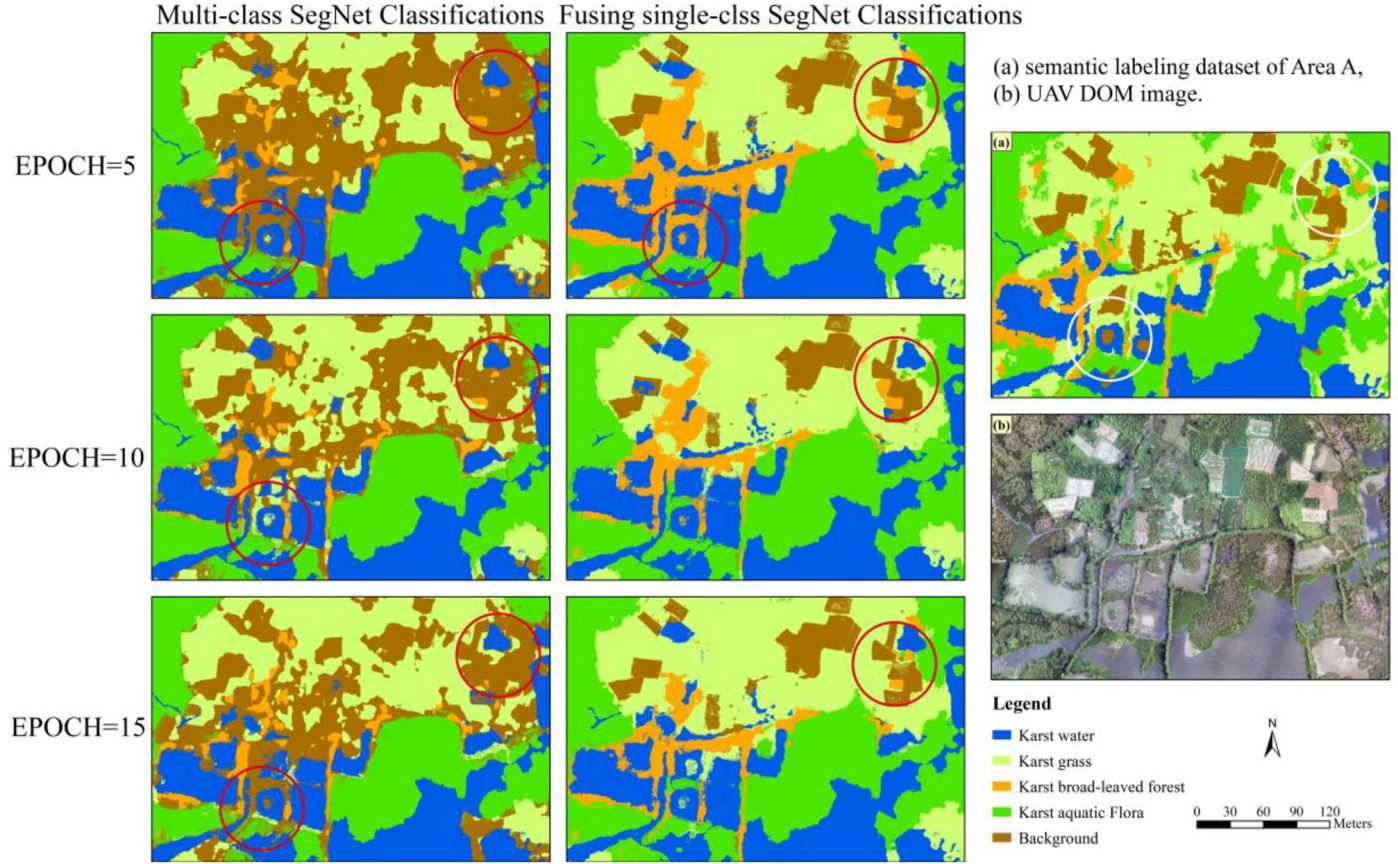
SegNet-based classifications for karst wetland vegetation with different EPOCH values. ArcGIS 10.6 software (http://www.esri.com/software/arcgis) was used to process and map the data by first author (T. D.) and corresponding author (B. F.). The DOM images and semantic labeling dataset was collected and created by T. D. and B. F.

Figure 7 showed karst vegetation classifications of Area A produced using the fusion of single-class and multi-class SegNet models with three EPOCH values, respectively. The classification results of each model in the Fig. 7 were marked with more observable errors in the red circles, and the correct vegetation type at the corresponding position is marked with white circles.

Figure 7 showed that the classification results of fusion of single-class and multi-class SegNet models become better with the increase EPOCH value. we found that *karst broad-leaved forest* and *karst grass* are better depicted by fusion of multiple single-class SegNet model than multi-class SegNet model in specific areas. For example, the predominance of *karst broad-leaved forest* of the study area was better represented around the karst river and ponds by single-class SegNet model without confusing between *karst grass* and *karst aquatic flora*. *Karst water* and *karst aquatic flora* were both better depicted using the SegNet models. Through visual comparison of classifications derived from multi-class SegNet model and fusion of multiple single-class SegNet model, the major visual difference in classifications is the other land use types, such as paddy field and vegetable fields in the study area, which were depicted mixed with *karst grass* due to the spectral similarity.

Table 4 displayed the classification accuracies (F1-score) for fusion of multiple single-class and multi-class SegNet models, respectively. We found that fusion of single-class SegNet model obtained higher F1-score than multi-class SegNet model for karst vegetation classifications, with the exception of *karst grass*. *Karst water* achieved over 0.86 F1-score using multi-class models. With the EPOCH value increasing from 5 to 15, The SegNet-based classification accuracy (kappa coefficient) improved 0.09, especially for fusion of single-class SegNet model increasing from 0.65 to 0.74. Moreover, the fusion of single-class SegNet model improved more classification accuracy (F1-score) for each wetland vegetation type in comparison of multi-class SegNet model. *Karst aquatic flora* obtained higher F1-score using the SegNet models with EPOCH 5 than that with EPOCH 10. The fusion of single-class SegNet model with EPOCH 10 for *karst grass* achieved the highest F1-score than the other EPOCH values. These results indicated that the EPOCH values of SegNet model have an effect on the classification accuracy of karst wetland vegetation. Comparing with single-class SegNet-based classifications (such as the *model-a, model-b*), the multi-class SegNet classifications achieved the lower F1-scoers for each vegetation type, but the differences in kappa coefficient were not statistically significant. These results demonstrated that the fusion of multiple single-class SegNet model could provide more accurate classifications for karst wetland vegetation, and the SegNet model with EPOCH 15 also outperformed the models with EPOCH 5 or 10 in vegetation classification.

**Table 4:** Classification accuracies of SegNet models with different EPOCH values.

Models	EPOCH	F1-Score				Kappa
		*Karst water*	*Karst grass*	*Karst broad-leaved forest*	*Karst aquatic flora*	
Multi-class SegNet model	5	0.88	0.61	0.46	0.71	0.59
	10	0.86	0.63	0.50	0.69	0.63
	15	0.91	0.68	0.52	0.75	0.68
Fusion of single-class SegNet model	5	0.91	0.59	0.52	0.80	0.65
	10	0.89	0.67	0.65	0.75	0.70
	15	0.92	0.66	0.66	0.81	0.74

### Single-class SegNet classifications with texture feature

**Figure 8:**
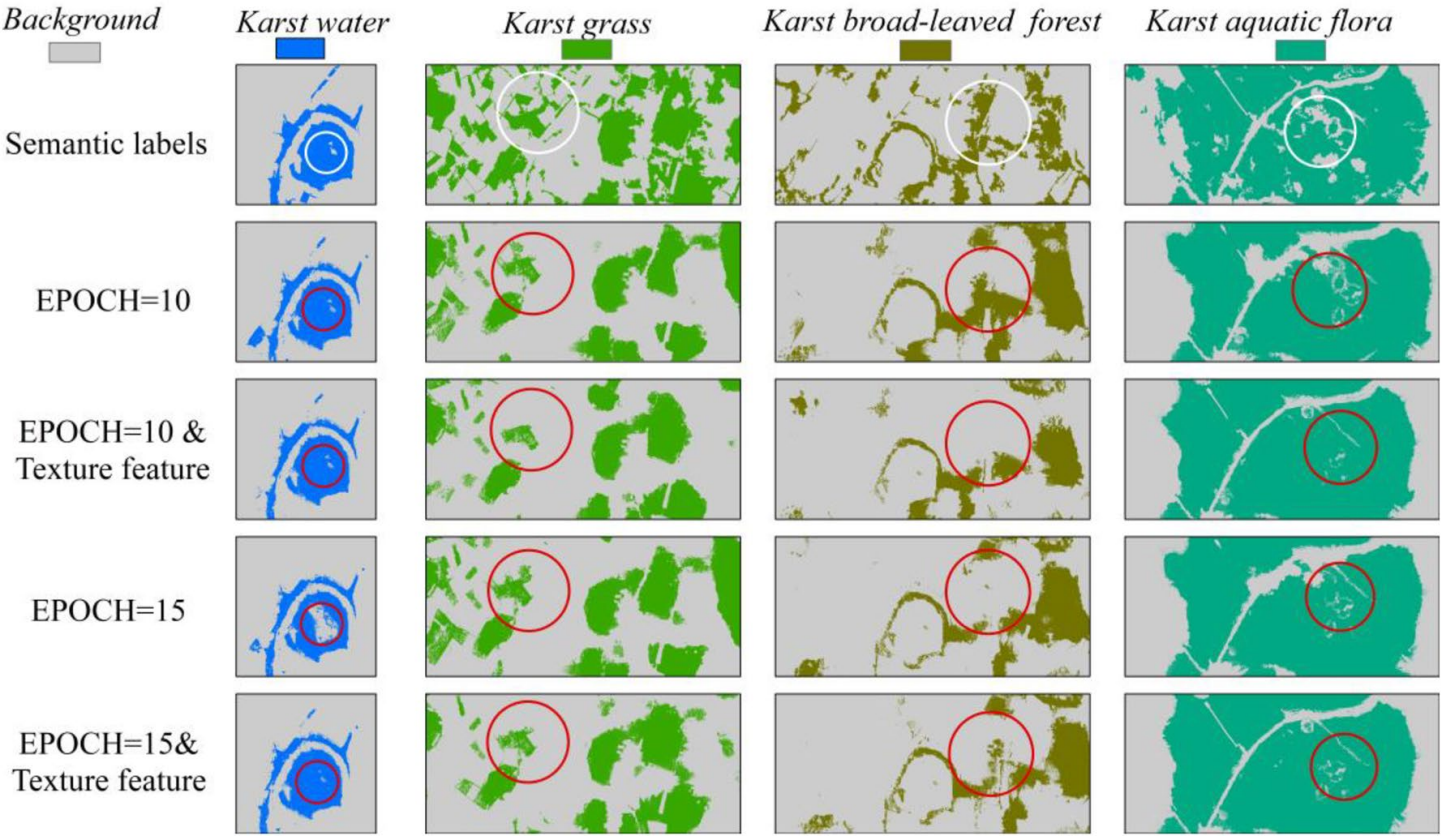
Single-class SegNet-based classifications for all karst wetland types with textural features. ArcGIS 10.6 software (http://www.esri.com/software/arcgis) was used to process and map the data by first author (T. D.). The semantic labeling dataset was collected and created by T. D. and B. F.

This study added textural features into the deep-learning image dataset of Area B for exploring the effect of texture feature on single-class SegNet classifications of karst wetland vegetation (Fig. 8). In order to comparison of visual difference in classifications, the typical area of each model classification was marked with red circles, and the correct vegetation type at the corresponding position was marked with a white circle from semantic labeling dataset.

The single-class SegNet model produced better visually accurate depictions of *karst water* and *karst aquatic flora* than *karst grass* and *karst broad-leaved forest*. The single-class SegNet model with EPOCH 15 and textural features accurately identified four karst vegetation. When the EPOCH value was set 10, the four models without textural features produced more visually accurate depictions than that with textural features. While the four models with textural features and EPOCH 15 provided the better depictions. Comparison of the models with texture features, the karst vegetation classifications were better depicted by the model with the EPOCH 15 than by the model with the EPOCH 10. The *model-w* with the EPOCH 15 produced the confusion between *karst water* and other land use types. The *model-b* with the EPOCH 10 produced the better depiction of *karst broad-leaved forest* than the model with EPOCH 15 or texture features (Fig. 8).

Table 5 showed the classification accuracies for four single-class SegNet models with or without texture feature in the area B. The single-class SegNet models for classifying karst vegetations achieved over 83% of F1-score, especially for the models with the textural features producing 0.97 F1-score and 0.94 kappa coefficient for *karst aquatic flora*. These results indicated that the single-class SegNet model provides the capability for monitoring spatial distribution of karst wetland vegetation using UAV images. The four models with the textural features improved the 4% F1-score and 6% kappa coefficient when comparing the model without texture feature classifications, respectively. The studies found that the classifications produced by combining single-class SegNet with texture features typically outperform only SegNet-based classifications. The texture feature enhances the capability of SegNet models in discriminating karst vegetations, and achieved over 0.92 F1-score for *karst aquatic flora*, *Karst Grass* and *karst water*. When the deep-learning dataset without texture feature, the SegNet model (*model-a*) with EPOCH 15 achieved over 0.95 F1-score for *karst aquatic flora*, which was higher than the other two karst vegetation types. When comparing the model classifications with the different EPOCH values, this paper found the following results: (1) the *model-a* with EPOCH 15 and texture features produced 0.97 F1-score for *karst aquatic flora*, which was 0.06 higher than this model with EPOCH 10 and without texture features. the *model-a* using just EPOCH 10 achieved 0.90 kappa coefficient, a decrease of 4% in comparison of the model with EPOCH 15 and texture features; (2) the *model-b* with EPOCH 15 improved F1-score and kappa coefficient to 0.86 and 0.72 in comparison of the model with the EPOCH 10, while the *model-b* using the combination of EPOCH 15 and texture features achieved higher classification accuracy for *karst broad-leaved forest*, reaching 0.88 F1-score and 0.78 kappa coefficient, an increase of 12% in comparison of the model with EPOCH 10; (3) the *model-g* with EPOCH 15 and 10 both achieved over 0.86 F1-score for *karst grass*. The *model-g* using the combination of EPOCH 15 and texture features improved F1-score and kappa coefficient to 0.92 and 0.83, an increase of 6 and 11% in comparison of the model with EPOCH 10, respectively; (4) the *model-w* with EPOCH 15 achieved higher classification accuracy for *karst water* than that with EPOCH 10, reaching 0.92 F1-score and 0.82 kappa coefficient. There were not differences in classification accuracy of *model-w* with EPOCH 10 and using the combination of EPOCH 10 and texture features. The *model-w* using the combination of EPOCH 15 and texture features improved F1-score and kappa coefficient to 0.95 and 0.90, an increase of 7 and 14% in comparison of the model with EPOCH 10, respectively. These results indicated that the single-class SegNet model could improve classification accuracy for karst vegetation types by using the combination of EPOCH 10 and texture features.

**Table 5:** Classification accuracy of single-class SegNet models with texture feature and different EPOCH values in area B.

Models	Karst vegetation	Textural feature	Epoch	Precision	Recall	F1-score	Kappa
*Model-a*	*Karst aquatic flora*	NO	10	0.95	0.92	0.91	0.87
			15	0.98	0.93	0.95	0.90
		YES	10	0.95	0.92	0.94	0.87
			15	0.97	0.97	0.97	0.94
*Model-b*	*Karst broad-leaved forest*	NO	10	0.81	0.87	0.83	0.66
			15	0.83	0.90	0.86	0.72
		YES	10	0.85	0.89	0.87	0.72
			15	0.87	0.89	0.88	0.78
*Model-g*	*Karst grass*	NO	10	0.92	0.82	0.86	0.72
			15	0.94	0.84	0.87	0.77
		YES	10	0.92	0.82	0.86	0.74
			15	0.95	0.89	0.92	0.83
*Model-w*	*Karst water*	NO	10	0.91	0.86	0.88	0.76
			15	0.93	0.91	0.92	0.82
		YES	10	0.93	0.91	0.92	0.84
			15	0.96	0.94	0.95	0.90

### Multi-class SegNet classifications with texture features

**Figure 9:**
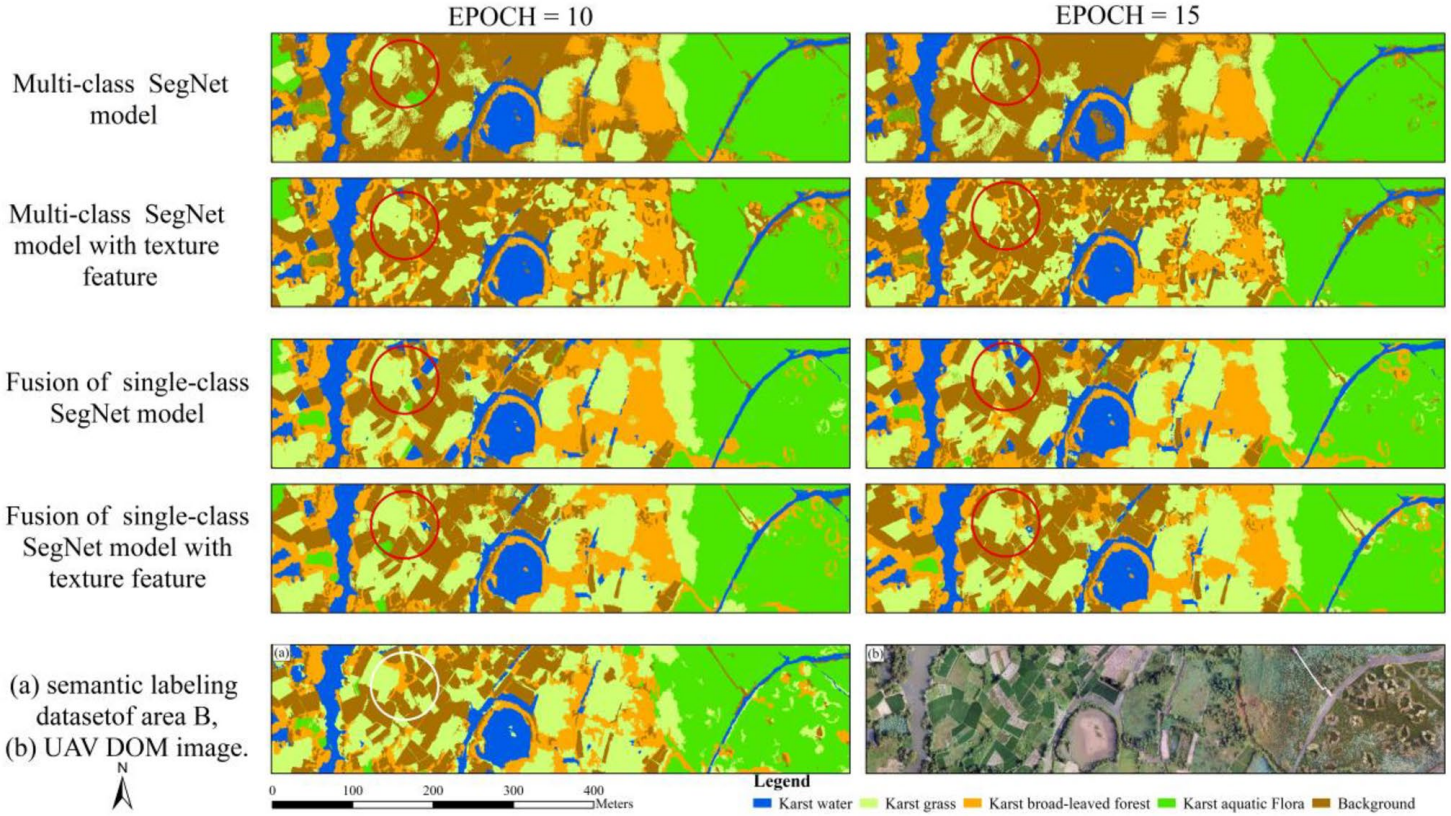
Classifications of multi-classification SegNet models with texture feature. ArcGIS 10.6 software (http://www.esri.com/software/arcgis) was used to process and map the data by first author (T. D.). The DOM images and semantic labeling dataset was collected and created by T. D. and B. F.

This paper attempted to evaluate the influence of textural features in classifying karst wetland vegetation between the multi-class SegNet model and fusion of single-class SegNet models in area B. In this study, four single-class SegNet models with the same EPOCH value were fused into multi-types classifications. The classification results were shown in Fig. 9. In order to comparison of visual difference in classifications, the typical area of each model classification was marked with red circles, and the correct vegetation type at the corresponding position was marked with a white circle from semantic labeling dataset.

The two multi-class SegNet-based classifications seen from Fig. 9 produced more visually accurate depictions of *karst water* and *karst aquatic flora* than *karst grass* and *karst broad-leaved forest* in the area B. The two multi-class SegNet models both overestimated classifications for *karst broad-leaved forest*, while the models underestimated classification of *karst grass* (Fig. 9). The multi-class SegNet model with EPOCH 10 produced more visually accurate depictions of *karst water* and *karst grass* than that with EPOCH 15. This corresponds with the use of the combination of EPOCH values and texture features. When using the combinations of EPOCH values and texture features, the SegNet model accurately identified karst vegetation, especially for *karst broad-leaved forest*, comparing the model without texture features. Comparison of the two multi-class SegNet model without texture features, several small patches of *karst grass* was identified by the SegNet model with the texture features. In the classifications derived from multi-class SegNet model without texture features, the eastern of the study area in the river valley was depicted the mixed area of *karst aquatic flora*, *karst broad-leaved forest* and other land cover types due to the spectral similarity. There were not visual differences in classifications produced by fusion of single-class SegNet models with the EPOCH values between 15 and 10. Compared to the fusion of single-class model classifications, this paper found that the models with texture features produced more visually accurate depictions than that without textural features, especially for *karst grass* and *karst broad-leaved forest*. Through visual comparison of multi-class SegNet-based classifications using the texture features and fusion of single-class SegNet classifications, we found that *karst grass* and *karst broad-leaved forest* are better depicted by multi-class SegNet-based classifications than by fusion of single-class SegNet classifications.While the fused SegNet-based classifications provided the better depictions of four karst vegetations than multi-class SegNet-based classifications with the texture features.

For multi-class SegNet-based classifications of karst vegetation in area B, the model with EPOCH 15 and 10 both achieved over 0.7 F1-score for all vegetation types (Table 6). *Karst water* was accurately classified in the model with the EPOCH 15, achieving 0.90 F1-score, which is higher than the other karst vegetation. The SegNet-based classifications using the EPOCH 15 for *karst broad-leaved forest* achieved 0.82 F1-score with an increase 4% in comparison of the model with EPOCH 10. The SegNet-based classifications for *karst grass* with the EPOCH 15 produced 0.78 F1-score, which was 0.07 higher than the classifications with EPOCH 10. When using the combinations of EPOCH values and texture features, the model produced the higher classification accuracy than that without texture features. The multi-class SegNet-based classifications using the combination of the EPOCH 15 and texture features achieved over 0.81 F1-score for all vegetation types. Meanwhile, the model achieved the highest classification accuracy for *karst water* (F1-score, 0.92), followed by *karst broad-leaved forest* (F1-score, 0.86). The SegNet-based classifications using the combinations of EPOCH 10 and texture features for *karst broad-leaved forest* improved F1-score to 0.86 with an increase 10% in comparison of the model with the EPOCH 10. The SegNet-based classifications for *karst aquatic flora* with the combinations of EPOCH 10 and texture features produced higher accuracy, achieving 0.87 F1-score, compared to using the combinations of EPOCH 15 and texture features. There were not differences in classification accuracy produced by the model between just with EPOCH 15 and with the combination of EPOCH 10 and texture features. The model using the combination of EPOCH 15 and texture features improved kappa coefficient to 0.76, an increase of 6% in comparison of the model with just the EPOCH 10. Meanwhile, the model with textural features improved 8% F1-score between in all karst vegetation classes when compared to the model with just the EPOCH 10, especially for *karst broad-leaved forest*.

**Table 6:** The classification accuracies of multi-class SegNet models with textural features and different EPOCH values.

Models	Textural feature	EPOCH	F1-Score				Kappa
			*Karst water*	*Karst grass*	*Karst broad-leaved forest*	*Karst aquatic flora*	
Multi-class SegNet model	NO	10	0.89	0.71	0.71	0.78	0.70
		15	0.90	0.78	0.73	0.82	0.73
	YES	10	0.89	0.79	0.76	0.87	0.75
		15	0.92	0.81	0.86	0.84	0.76
Fusion single-class SegNet model	NO	10	0.95	0.76	0.76	0.81	0.76
		15	0.95	0.77	0.73	0.87	0.81
	YES	10	0.97	0.77	0.81	0.81	0.81
		15	0.97	0.79	0.83	0.83	0.84

For the karst vegetation classifications of area B using the fusion of single-class SegNet models, the model with EPOCH 15 and 10 both achieved over 0.71 F1-score and 0.76 kappa coefficient for all vegetation types. There were not differences in classification accuracy produced by the models with the EPOCH values between 10 and 15. The SegNet-based classifications for *karst water* produced 0.95 F1-score, which was higher than the other vegetation types. The classifications using the EPOCH 15 achieved 0.87 kappa coefficient, an increase of 6% in comparison of using the EPOCH 10. When using the combinations of EPOCH values and texture features, the model produced the higher classification accuracy for all vegetation types, except for *karst aquatic flora*. The fused SegNet-based classifications using the combination of the EPOCH 15 and texture features achieved over 0.79 F1-score for all vegetation types. The fused model achieved the highest classification accuracy for *karst water* (F1-score, 0.97) when utilizing the combination of the EPOCH 10 and texture features (Table 6). There were not differences in classification accuracy produced by the fused model with the combination of texture features and EPOCH values increasing from 10 to 15. The fused model with EPOCH 15 achieved 0.87 F1-score for *karst aquatic flora*, which was higher than the fused model with the combination of texture features and EPOCH 15. In addition, the fused model using the combination of EPOCH 15 and texture features improved 8% kappa coefficient compared to the model with EPOCH 10.

Comparison of SegNet-based classifications in area B, this study found that multi-class SegNet model produced the higher F1-score than the fused model for all vegetation types, except for *karst water*. Whereas the fused model achieved 0.84 kappa coefficient with an increase of 14% in comparison of multi-class model with EPOCH 10.

### Comparison of object-based RF and fusion of multiple single-class SegNet classifications

This paper found that fusion of multiple single-class SegNet model with the combination of EPOCH 10 and texture features produce the best performance in mapping karst vegetation. Table 7 displayed the overall accuracies for fused SegNet-based and object-based RF classifications in area A and area B, respectively. There were not differences in classification accuracies between object-based RF and the fused SegNet models, both achieving over 0.87 overall classification accuracy. User’s and producer’s accuracies were summarized for each vegetation types in four classification scenarios (Table 7). Object-based RF classifications achieved over 90% producer’s accuracy for all categories. *Karst water* was accurately classified by object-based RF and the fused SegNet models, and achieved over 0.95 user’s and producer’s accuracies, respectively. The object-based RF classifications achieved over 0.90 producer’s accuracies for *karst broad-leaved forest* and *karst aquatic flora*, which were higher than the fused SegNet-based classifications. The producer’s accuracy of *karst grass* was consistently over 0.90 for the two models. The two models achieved over 0.89 user’s accuracy for all categories, with the exception of *karst grass*. The object-based RF classifications achieved higher user’s accuracy for *karst grass* than the fused SegNet-based classifications.

**Table 7:** Comparison of classification accuracies between object-based RF and fusion of single-class SegNet models.

Models	Accuracy evaluation	*Karst water*	*Karst grass*	*Karst broad-leaved forest*	*Karst aquatic flora*
Object-based RF model	Producer accuracy	0.95	0.90	0.95	0.90
	User accuracy	0.96	0.94	0.95	0.89
	OA	0.92			
	Kappa	0.90			
Fusion of single-class SegNet model	Producer accuracy	0.95	0.95	0.75	0.75
	User accuracy	1.00	0.68	0.94	0.94
	OA	0.87			
	Kappa	0.84			

## Discussion

The single-class SegNet-based classifications achieved over 0.83 F1-score, and the fusion of multiple SegNet-based classifications achieved 87% overall accuracy for all vegetation types. These results demonstrated that the SegNet deep-learning algorithm could provide the high-precision classifications of karst wetland vegetation using the high spatial resolution UAV images. The single-class SegNet model achieved an improvement in classification accuracy (F1-score values) of 5–21% compared to multi-class SegNet-based classification, and produced over 0.88 F1-score for karst wetland vegetation, this was consistent with reported studies that the deep-learning algorithm can accurately identify simple and pure ground objects, such as river^47^ and artificial building^48^. In this paper, we found several limitations of SegNet-based deep-learning classifications, including small or isolate patches of *aquatic flora* vegetation without identifying, and classification errors between mixed pixels of vegetation types, such as *Karst Grass* and *Karst Broad-leaved Forest*. The main reason of these problems is that we only used the UAV RGB DOM image and textural features, and do not provide enough useful information. Previous studies have reported that wetland vegetation usually has the high spectral similarity, and DSM data with the vertical structure information^49^, and spectral indexes^50^ could enhanced spectral separability of wetland vegetation. Scholars can attempt to use combinations of UAV DOM image with more spectral bands, vegetation and water spectral indexes, DSM image and other data source (such as LiDAR point cloud Data) to classifying karst wetland vegetation in the future. In addition, the optimizing image algorithm proposed by this paper could eliminate the stitching traces of SegNet-based classifications, and improve classification performance of karst wetland vegetation. The post-processing algorithm is also applied to other deep-learning classification models.

Model fusion or model combination has been an approach to improve classification performance of single model. Zhao & Liu proposed a CNN-based feature extraction from the MINST dataset and algebraic fusion of multiple classifiers trained on different feature sets^51^. Classifier fusion achieved 98% overall accuracy. This paper is different from the reported study, and the single-class SegNet model was used to discriminate each land cover type, then an approach of maximum probability was utilized to fuse single-class SegNet-based classifications for mapping all karst vegetation types in the study area. Comparison of the accuracy differences between fused single-class and multi-class SegNet-based classifications, this paper found that the fused SegNet-based classifications outperformed the multi-class SegNet classifications, and produced 0.81 kappa coefficient with an improvement of 11%. The fused SegNet model also improved classification accuracy of mixed pixels of vegetation patches of *karst broad-leaved forest* and *karst aquatic flora*, respectively. These conclusions demonstrated that fusion of multiple single-class SegNet models improves the performance for identifying karst vegetations using UAV images. The fusion of multiple SegNet-based binary classification (one SegNet for each land cover types instead of one SegNet for all land cover types) has some benefits such as higher accuracy, quick training and use of small dataset.

This paper examined the effect of the SegNet model with different EPOCH values on mapping karst wetland vegetation, and revealed that the SegNet model using the EPOCH 15 achieve higher classification accuracy (kappa coefficient) than the EPOCH values with 10 or 5. The single-class SegNet model with the EPOCH 15 in area A, achieved an improvement in classification accuracy of 6 ~ 14% for classifying karst wetland vegetation, respectively, compared to the models with EPOCH 5. The fused SegNet-based and multi-class SegNet-based classifications with the EPOCH 15 both achieved higher kappa coefficient in comparison of the model with the EPOCH 5. These results confirmed that the SegNet model with the EPOCH 15 was suitable to karst wetland vegetation classification. In addition, this study found that when the deep-learning UAV image dataset added the textural features, the SegNet models improved the classification accuracy of karst vegetation with an improvement in kappa coefficient of 7–14% comparison of image dataset with only color features. This result was consistent with reported studies that found textural information can help improve the spectral separability of wetland vegetation^52–54^. When capturing UAV images, the weather conditions can significantly change the color intensity or variations in an image. The difference in illumination and altitude of the sun leads to a change in the appearance of vegetation patches captured by the RGB sensor. Some scholars used a color correction approach to improve the applicability of RGB images from different times and light conditions for consistent wetland mapping^55^. The single-class SegNet models with texture features improved over 5% F1-score compared to the model without texture features. The multi-class SegNet model using the combination of EPOCH 10 and texture features improved about 15% F1-score for *karst broad-leaved forest*. This demonstrated that texture features contained in the multi-view data could provide an effective information complement for color intensity of UAV image to karst wetland vegetation classification.

## Conclusions

This paper proposed a novel approach to classify karst wetland vegetation in the Huixian National Wetland Park using a fusion of multiple SegNet deep-learning network with UAV RGB images. We evaluated the performance of multi-class SegNet model and fusion of multiple single-class SegNet models on karst wetland vegetation mapping, and demonstrating that the feasibility of fusing multiple single-class SegNet classifications using the maximum probability approach. This paper examined the effectiveness of the post-classification algorithm developed by this paper for eliminating the splicing traces caused by model classification of SegNet network. Finally, we explored the effect of different EPOCH values and textural features on karst wetland vegetation mapping. The study confirmed that fusion of multiple SegNet-based binary classification outperforms multi-class SegNet-based classification, and achieved over 87% overall accuracy. The iterative number of model training (EPOCH values) and textural features have different effects on mapping vegetation of karst wetland. Texture features are an effective information complement for the UAV RGB images. The optimized post-classification algorithm could eliminate splicing traces, and improved classification accuracy of SegNet model.

The limitations of this paper did not consider the phenological differences, and the UAV RGB image with its textural features did not provide enough information to distinguish vegetation communities. We only use a SegNet deep-learning network for vegetation classification. In future research, we will attempt to combine multi-temporal UAV multispectral, hyperspectral images and LiDAR point cloud data, and use fusion of different deep-learning network or Stacking ensemble learning to classify vegetation communities of karst wetland.

## Data Availability

The datasets used and/or analysed during the current study are available from the corresponding author on reasonable request.

## Code availability

The custom code used and/or analysed during the current study are available from the corresponding author on reasonable request.
